# Debye Temperature and Quantum Diffusion of Hydrogen in Body-Centered Cubic Metals

**DOI:** 10.1021/acsomega.1c05902

**Published:** 2022-03-01

**Authors:** Vladimir Vykhodets, Olga Nefedova, Tatiana Kurennykh, Sviatoslav Obukhov, Evgenia Vykhodets

**Affiliations:** † Institute of Metal Physics UB RAS, 18 S. Kovalevskaya Street, Ekaterinburg 620108, Russia; ‡ Institute of Engineering Science UB RAS, 34 Komsomolskaya Street, Ekaterinburg 620049, Russia; § Ural Federal University Named After the First President of Russia B. N. Yeltsin, 19 Mira Street, Ekaterinburg 620002, Russia

## Abstract

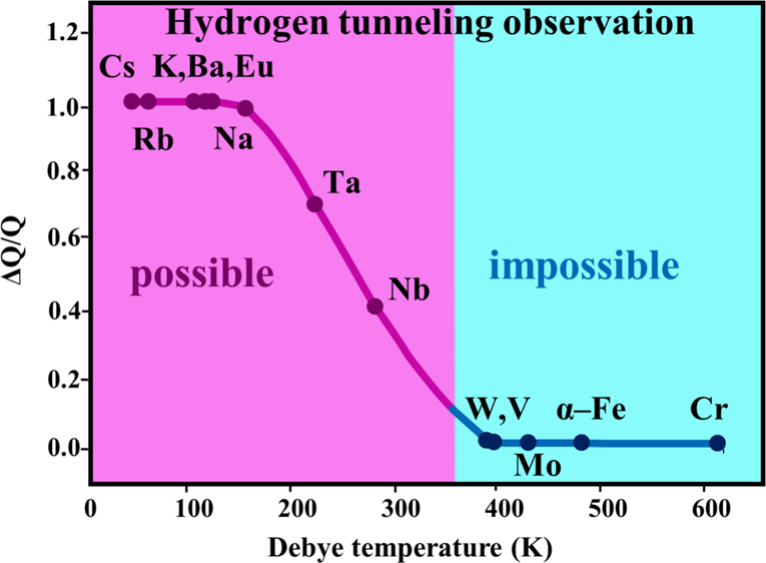

Diffusion of deuterium in potassium is studied herein. Mass transfer is controlled predominantly by the mechanism of overbarrier atomic jumps at temperatures 120–260 K and by the tunneling mechanism at 90–120 K. These results together with literature data allowed us to determine conditions under which the quantum diffusion of hydrogen in metals can be observed, which is a fundamental problem. It is established that in metals with a body-centered cubic lattice tunneling can be observed only at temperatures below the Debye temperature θ_D_ solely for metals with θ_D_ < 350 K. Predictions are made for metals in which quantum diffusion of hydrogen can be experimentally registered. Metals for which such results cannot be obtained are specified as well. Among them are important engineering materials such as α-Fe, W, Mo, V, and Cr.

## 1 Introduction

Hydrogen, the lightest chemical element, is involved in many natural and technological processes, including those at cryogenic temperatures. In view of this fact, many studies have been devoted to its quantum diffusion in metals, and their results are of high relevance in quantum physics, low-temperature technologies, theories of diffusion, and astrophysics. The issues of tunneling are mainly studied theoretically, and the main problem of this research trend is the quite scarce experimental database. The situation is somehow paradoxical because tunneling and overbarrier atomic jumps are the only existing mechanisms of atomic migration in solids; yet, experimental data on quantum diffusion of hydrogen have been obtained only for three metals: in Nb and Ta for protium^1,2^ and in Na for deuterium.^3^ Tunneling was also observed when hydrogen diffusion occurred on the metal surface, not in the bulk, and in this case, experimental data were gained for many metals.^4−8^ In all cases, observation of quantum diffusion was based on the registration of bends on the temperature dependences of diffusion coefficients *D*(*T*) in the coordinates log *D* – 1/*T*. Bends are caused by the competition of two mechanisms of atomic migration: mass transfer mainly goes by the quantum migration mechanism below the bend temperature *T*_b_ and by the classical mechanism above *T*_b_.^9,10^ In what follows, the bend will be characterized by a parameter Δ*Q*/*Q*, where *Q* is the energy of diffusion activation for *T* > *T*_b_ and Δ*Q* is its change at the inflection point. All metals for which data on the quantum diffusion of hydrogen were obtained possess a body-centered cubic lattice, bcc.

To describe the quantum diffusion of hydrogen in metals, several approaches were applied: the small polaron model of Flynn and Stoneham,^10−14^ Feynman path-integral molecular dynamics model,^15−17^ density functional theory (DFT),^12,13,15,16^ semiclassical transition state theory,^13^ centroid and ring polymer molecular dynamics,^18^ nonequilibrium statistic thermodynamics,^9^ and others. The interpretation of bends on the *D*(*T*) dependences in Ta and Nb^9,10,12^ was definitely successful. Quantum theories of diffusion, unlike classical theories, not only succeeded in explaining the bends but also made it on a quantitative level. Historically, the bends had first been explained by the polaron model;^10,11^ later, they were explained using the model based on non-equilibrium statistical thermodynamics. For example, the work^9^ reported on the determination of activation energies for protium diffusion in Nb, the values were 0.07 and 0.10 eV for quantum and classical migration mechanisms, respectively. The corresponding experimental values are 0.068 and 0.106 eV.^1^ The work^12^ showed first-principles DFT calculations of diffusion activation energies performed for both migration mechanisms, which demonstrated excellent agreement with experimental data for protium in Nb and Ta. At the same time, theoretical studies presented no explanation of the observation of quantum hydrogen diffusion only in few bcc metals, and the possibility of observation of hydrogen tunneling in other bcc metals was not questioned. For instance, in ref (18), the data on the quantum diffusion of tritium in α-Fe were calculated, and such an approach was considered pertinent to taking into account the timeliness and complexity of experiments on tritium diffusion in metals at cryogenic temperatures.

At present, there are no ways of predicting if the bend on the *D*(*T*) dependences can be observed, and, accordingly, whether quantum diffusion is possible in this or that bcc metal, as well as what value of Δ*Q*/*Q* can correspond to this bend. However, this task is a topical one, in particular, for many applications because at cryogenic temperatures, coefficients of classical and quantum diffusion of hydrogen can differ by several orders. The objective of the current work was to develop such an approach, so one more metal, potassium, was studied to expand the experimental database on quantum diffusion in bcc metals, and, second, we analyzed the dependences *T*_b_(θ_D_) and , where θ_D_ was the Debye temperature of a metal. The choice of the Debye temperature as an argument of the dependences characterizing quantum diffusion is substantiated by the fact that, for many properties, θ_D_ indicates an approximate boundary below which quantum effects manifest themselves. Potassium was chosen because its θ_D_ = 100 K is the lowest among other bcc metals for which data on hydrogen tunneling are known. Moreover, the range of θ_D_ values for which data on the hydrogen tunneling were obtained is expanded by a factor of 1.5, which enhances the reliability of regularities that can be established when studying dependences *T*_b_(θ_D_) and .

As few as three techniques were employed in the worldwide studies of quantum hydrogen diffusion. The results on protium tunneling in Nb and Ta were obtained 40 years ago using methods based on the Gorsky effect^1^ and NMR measurements of spin–lattice relaxation.^2^ They allowed measuring *D* values higher than 10^–13^ m^2^ s^–1^. Data on the quantum diffusion of deuterium in Na were gained with an accelerator technique, nuclear reaction analysis online (NRAOL).^3^ It employs Fick’s diffusion equations and measurements of deuterium concentration using the nuclear reaction ^2^H(d,p)^3^H. For NRAOL, the measured *D* values were several orders lower compared to the competing methods, from 10^–17^ to 10^–12^ m^2^ s^–1^,^19^ which made it possible to expand the experimental database. At the same time, NRAOL has a disadvantage—it can be applied to the investigation of only one hydrogen isotope, deuterium. For comparison, the technique based on the Gorsky effect was used in studies for all three isotopes in V, Nb, and Ta at cryogenic temperatures.^1^ For the objective of our work, the NRAOL technique is the best fit; using it, particular data on *D*(*T*) for deuterium in another alkaline metal Na have already been obtained.^3^ Methods of sample preparation, processing of initial data, and other procedures for Na and K were identical.

## 2 Experimental Methods

A distinguishing feature of NRAOL is time phasing of three stages of diffusion experiments: formation of a diffusion source, diffusion annealing, and measurement of concentration profiles *c*(*x*, *t*). In our case, *c* is the deuterium concentration at a depth *x* in sample, and *t* is the experimental timing. Accordingly, in NRAOL, the sample is placed into a vacuum chamber of the accelerator setup and continuously irradiated with deuterons; at the same time, it undergoes isothermal diffusion annealing and the products of reaction ^2^H(d,p)^3^H, proton spectra, are registered. It is just simultaneous operations that provide the principal possibility for NRAOL to measure the diffusion coefficients at any temperatures, including cryogenic temperatures. As for the range of *D* values measured with an appropriate accuracy, it is controlled by the section of nuclear reaction ^2^H(d,p)^3^H and the constants characterizing the interaction of accelerated deuterium with substances. Experiments were performed on a 2 MV Van de Graaff accelerator. The energy of deuterons of the incident beam was 650 keV, beam diameter was 2 mm, and current strength of the beam was kept constant within 10%. The statistical error in the determination of radiation dose was about 1%. The flat sample surface was perpendicular to the axis of the incident beam. The internal source of deuterium diffusion, which formed upon the irradiation of potassium with deuterons, was located inside the sample at a depth of 18.3 μm, which was determined using the SRIM program.^20^ To register protons, a silicon surface-barrier detector was employed, the angle of registration being 160°. Mathematical processing of primary data was conducted via the procedure of comparing spectra from the samples under study with the reference spectrum from the deuteride ZrCr_2_D_0.12_ with a constant-in-depth deuterium concentration.

Alkali metals easily become oxidized in air and in as-supplied state are stored in glass-vacuumed ampules. Therefore, to break up ampules with potassium, a box with a low content of oxygen and water vapor, no more than 0.5 ppm, was engaged. In the box, potassium was transferred into a transport container, where it was sealed hermetically. Then, the container was placed into a chamber of the accelerator setup and opened under conditions of a high vacuum. To heat and cool samples, a resistive furnace and flow-through liquid nitrogen were employed, the temperature being the lower boundary of diffusion investigations. Devices for assembling samples and their annealing are described in the Supporting Information section and in works.^3,21,22^ Examinations using methods of nuclear reactions (NRAs) showed that the samples were of purity sufficient for diffusion experiments, the oxygen content did not exceed 0.2 at. % and no nitrogen or carbon impurities were detected. The sample temperature upon diffusion annealing was kept constant within ±1 K.

## 3 Results and Discussion

**Figure 1:**
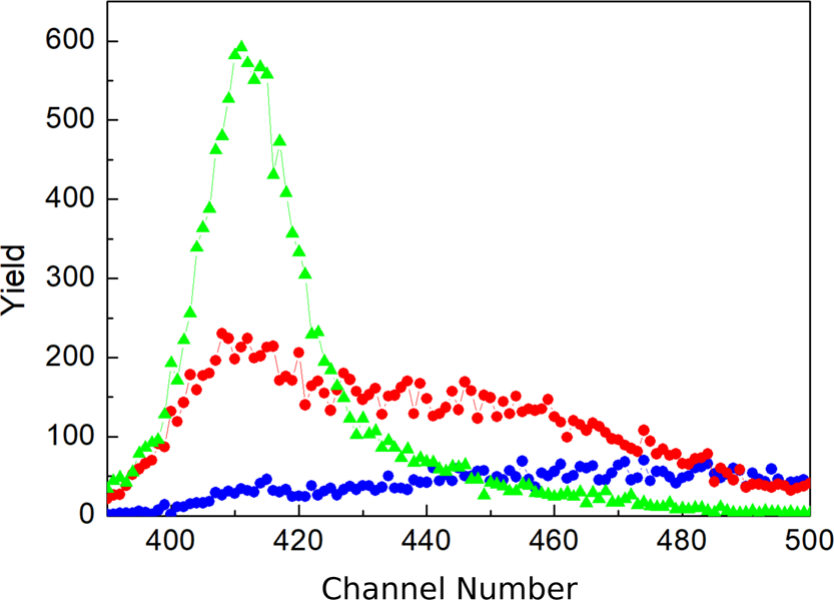
Effect of diffusion annealing temperature *T* on spectra of the nuclear reaction ^2^H(d,p)^3^H product for potassium samples annealed at *T* = 250 K, green; *T* = 170 K, red; and *T* = 90 K, blue. Radiation doses are close.

Figure 1 shows the selected primary data used in the NRAOL technique to determine diffusion coefficients of deuterium. The “yield” values on the *Y* axis are proportional to the number of protons detected from the ^2^H(d,p)^3^H reaction, whereas the “channel number” values on the *X* axis are proportional to proton energy, so the data in Figure 1 are similar to energy spectra. These spectra in the NRA and NRAOL techniques contain information on atomic concentration profiles *c*(*x*, *t*). Figure 1 shows that the spectra’s outline and height depend significantly on the diffusion annealing temperature for the samples. This indicates that the NRAOL technique is highly sensitive to *D* values. The content of deuterium in the diffusion zones under the study was less than 0.4 at. %.

To determine *D* values from the concentration profiles *c*(*x*, *t*), two solutions of the diffusion equation with the boundary conditions set in the experiment were used. Both took into account the specificity of the NRAOL technique that was employed to continuously irradiate the sample with deuterons. Upon irradiation, inside the sample at a depth *x*_0_, there takes place formation on an internal diffusion source from which deuterium atoms diffuse toward the irradiated surface of the sample and backward. In the frame of NRAOL, the *c*(*x*, *t*) profiles are measured only at *x* < *x*_0_. The solutions of the diffusion equation that were used in the work are obtained with the following assumptions: the flow of deuterons from the accelerator do not depend on the irradiation time *t*; deuterium atoms are not present in the sample prior to irradiation; the sample thickness *l* ≫ *x*_0_; diffusion flow of deuterium atoms through the irradiated sample surface is equal to 0; and the diffusion coefficient does not depend on the coordinated *x*. These conditions were monitored with relevant accuracy.

When solving diffusion equations, two scenarios which corresponded to the formation of two different-in structure internal diffusion sources were considered: solid solutions of deuterium in potassium K–D (scenario 1) and potassium deuteride KD in equilibrium with solid solution K–D (scenario 2). For scenario 1, the deuterium diffusion was carried out from an infinitely thin layer to the irradiated and non-irradiated sample surfaces, while the deuterium concentration at a depth *x* = *x*_0_ increased with the time *t* of irradiation. For scenario 2, deuterium concentration c* at a depth *x* = *x*_0_ did not depend on the time *t*, it was equal to the deuterium concentration in a solid solution in equilibrium with deuteride.

For the first scenario, the analytical solution of the boundary value problem was used^3^
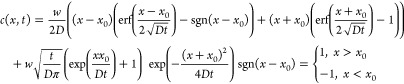
1Here, *w* is the flux deuterium ions during sample irradiation.

For the second scenario, the analytical solution was used^3^
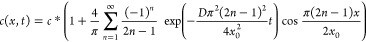
2

We supposed that the first scenario was more probable that at elevated temperatures, whereas the second scenario was more probable at low ones. Upon irradiation, there occurs the implantation of deuterium ions into a depth *x*_0_ and, simultaneously, migrating deuterium atoms leave the zone of the internal diffusion source into other parts of the sample bulk. In competition of these two processes, the necessary conditions for the deuteride formation is the low value of the diffusion coefficient for deuterium; hence, there can be two expressions for *c*(*x*, *t*). The results of this work confirmed the following: the experimental data on *c*(*x*, *t*) are satisfactorily described by eq 1 at *T* ≥ 200 K and by eq 2 at *T* < 200 K. In the research on deuterium diffusion in Na, temperatures for the application of eqs 1 and 2 were almost the same.

**Figure 2:**
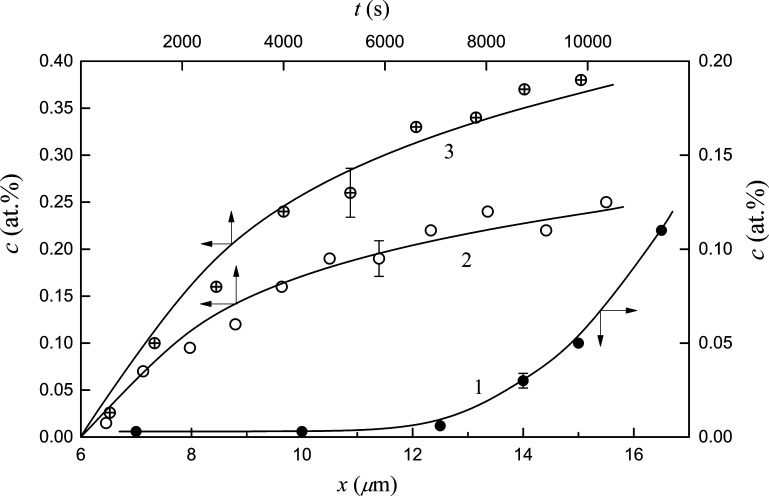
Deuterium concentration *c* as a function of annealing time *t* and depth *x* in potassium samples. Points show experimental data and lines and analytical dependences according to eqs 1 and 2. The *D* values in eqs 1 and 2 are considered independent from *t* and *x*. Curves 1, 2, and 3 correspond to annealing temperatures *T* = 113, 133, and 202 K, respectively; curve 1 corresponds to annealing time *t* = 11 050 s; curves 2 and 3 correspond to depths *x* = 16.5 and 7 μm, respectively.

**Figure 3:**
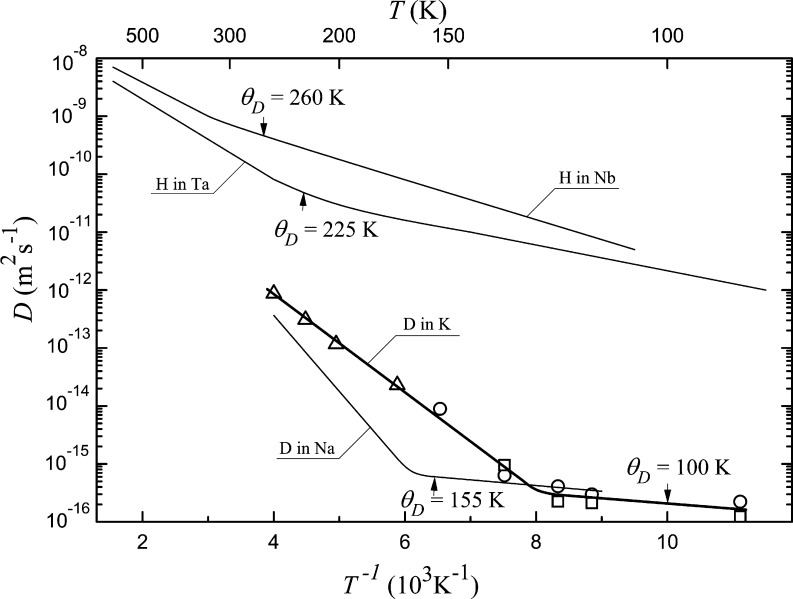
Temperature dependences of diffusion coefficient *D* for protium in Nb^1^ and Ta^1^ and deuterium in Na^3^ and K. Arrows show Debye temperatures of metals. Points demonstrate experimental results on deuterium diffusion in K: triangles are obtained with the use of eq 1 and data on *c*(*t*); circles and squares are obtained using eq 2 and data on *c*(*t*) and *c*(*x*), respectively. Mean-square errors in the determination of diffusion coefficients *D* are less than the size of a point.

In Figure 2, dependences *c*(*x*) and *c*(*t*) are shown for fixed annealing times and depths inside the sample, respectively. Deviations of experimental points from the analytical dependences in Figure 2 are mostly less than mean-square errors in the measurements of the deuterium content using the NRA method. The measurement errors are shown in Figure 2 for the three experimental points; in all the other cases, the errors in investigating the *c*(*t*) and *c*(*x*) dependences are also around 10%. Equations 1 and 2 were derived supposing that the diffusion coefficient for deuterium does not depend on coordinate *x* in the sample, and the results in Figure 2 purports the fulfilment of this condition despite the fact that upon applying NRAOL, in the sample radiation defects form, which can, in a common cases, affect the *D* values.^19^ However, in K, radiation defects were annealed at far lower temperatures^23^ than the range of diffusion experiments and therefore could not influence the deuterium diffusion. At all temperatures, the diffusion coefficients were determined from the dependences *c*(*t*), *T* ≤ 150 K, and from the data on *c*(*x*) as well. The first method is more precise, yet, in two-phase samples, the rate of mass transfer can be restricted, along with diffusion, by the rate of reaction at interphase boundaries and the correctness of measurements is supported by the equality of *D* values found by the two methods. As is seen from Figure 3, these values coincide within the statistical error.

The bend in the dependence *D*(*T*) in Figure 3 indicates the quantum mechanism of deuterium diffusion in K below the bend temperature. The energy of diffusion activation above the bend temperature made up 0.16 eV, while below, it was close to 0. In Figure 3, along with the results for potassium, literature data on bends on the *D*(*T*) dependences for bcc metals are shown. For the systems Nb–H, Ta–H, Na–D, and K–D, the bend temperatures are 250, 229, 160, and 120 K and Debye temperatures 260, 225, 150, and 100 K, respectively. The bends are seen to occur in the vicinity of Debye temperature for all metals. Thus, the coefficient of hydrogen diffusion in bcc metals is a property for which the Debye temperature is an approximate boundary between quantum and classical behavior, which is a characteristic of the other properties that depend on atomic vibration spectra, say heat capacity. In theoretical works, different opinions on the relation of *T*_b_ and θ_D_ are presented. In ref (9), using propositions of nonequilibrium statistical thermodynamics, it is shown that the inflection takes place near θ_D_. In the models based on centroid and ring polymer molecular dynamics,^18^ the values of *T*_b_ and θ_D_ are independent.

As is seen in Figure 3, bends on the dependences *D*(*T*) were found only for hydrogen in metals with θ_D_ ≤ 260 K. At the same time, a low Debye temperature did not serve as a sufficient condition for the observation of quantum diffusion: bends were absent in the cases of diffusion of deuterium and tritium in Nb and Ta.^1^ Hence, there exist two types of the bcc system with θ_D_ ≤ 260 K: with and without bends on *D*(*T*). This principal distinction of quantum properties of systems can be substantiated by the difference in trajectories of hydrogen atoms upon diffusion in a bcc lattice. Our hypothesis is that the bends on *D*(*T*) dependences will be observed in cases, where tetrahedral interstitials are equilibrium positions for hydrogen atoms. On the contrary, the bends will not be observed if the equilibrium positions are octahedral interstitials. This hypothesis is based on the following reasonings. The difference in the distance between the nearest tetrahedral interstices in the bcc lattice is 1.5 times less between the octahedral ones and it is sufficient for the values of tunnel matrix elements for two types of systems to differ by several orders.^12^ In the classical approximation, equilibrium positions for hydrogen atoms in Nb, Ta, W, and α-Fe, according to the DFT calculations,^12,24,25^ are tetrahedral interstices, and octahedral ones do not take part in elemental migration acts. In models that take into account quantum effects, diffusion paths have octahedral interstitials^17^ and in these cases, bends on the *D*(*T*) dependences are absent because the tunneling of hydrogen atoms between equilibrium positions has a low probability.

**Figure 4:**
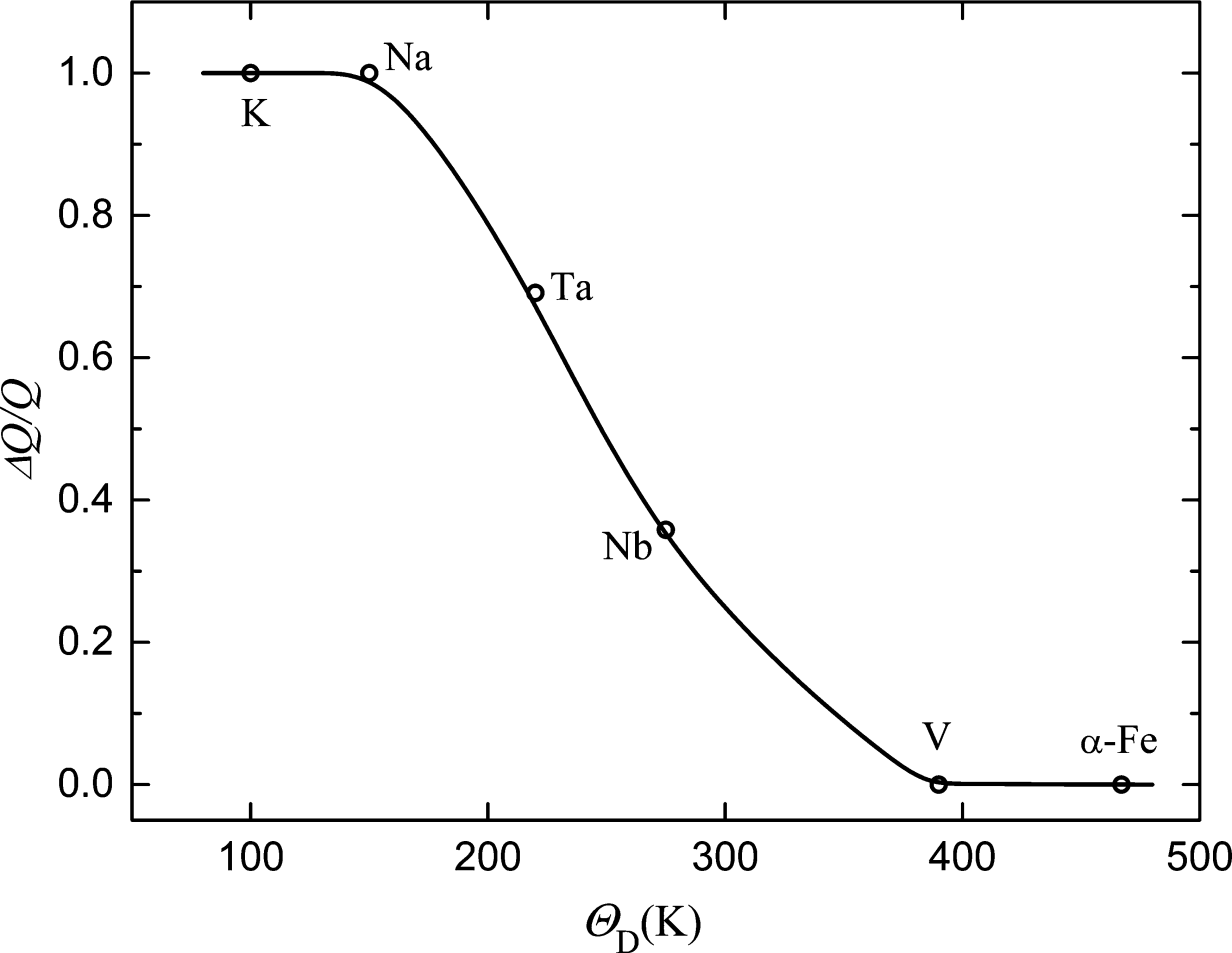
Dependence of the relative change of diffusion activation energy Δ*Q*/*Q* on Debye temperature θ_D_ for bcc metals.

Figure 4 shows the dependence , in which all the data on quantum diffusion of hydrogen in bcc metals and results for three hydrogen isotopes in V^1^ and protium in α-Fe^26−28^ are taken into account; yet, those for systems Nb–*D*(*T*) and Ta–*D*(*T*)^1^ are omitted. The results for V and α-Fe are obtained as for metals with θ_D_ > 260 K and in the temperature ranges including Debye temperature. The data for Nb–*D*(*T*) and Ta–*D*(*T*) were excluded from the dependence  because the bends in these systems are absent due to other reasons possibly because their atomic diffusion pathways differ from those for Nb–H and Ta–H. The values of Δ*Q*/*Q* are seen to be close to 1 for metals with θ_D_ < 160 K and with increasing θ_D_, they gradually decrease, so that, within the stated accuracy of the diffusion experiment, bends on the dependences *D*(*T*) can be registered only for metals with θ_D_ < 350 K. Analyzing with the allowance for quantitative characteristics of the  dependence and Debye temperatures for bcc metals, it is possible to conclude that a small body of experimental data on hydrogen tunneling in bcc metals can be caused by the high values of the Debye temperature for many bcc metals. Moreover, from the results of this work, the database on the quantum diffusion of hydrogen in bcc metals will not be expanded in the near future. One can expect only one or two new results: for Ba and Eu, whose Debye temperatures are about 120 K. There are grounds to assume that data on the deuterium quantum diffusion in Ba can be obtained with the NRAOL technique because the *D* values for the alkali metals Na and K and those for alkaline-earth element Ba are close to each other near the Debye temperatures. For Ba, there are no results on hydrogen diffusion at low temperatures, and information on the level of *D* values at 120 K is gained via extrapolating the data for the range from 20 to 600 °C.^29^ For Eu, experimental data on hydrogen diffusion are totally unavailable and, therefore, to make a prediction for applicability of one or another technique for diffusion experiments at cryogenic temperatures is hardly possible. Besides Ba and Eu, two more bcc metals have the value of θ_D_ < 350 K, they are Cs and Rb with θ_D_ equal to 56 and 40 K, respectively. Nowadays, there is no existing technique known to allow diffusion studies of hydrogen in metals at temperatures these low.

## 4 Conclusions

Thus, the results of the work are data on the temperature dependences of diffusion coefficients *D*(*T*) for deuterium in K. At temperatures from 250 to 120 K, the mechanism of overbarrier atomic jumps dominates in the process of mass transfer, whereas below 120 K, it is the mechanism of tunneling. At present, the database on hydrogen diffusion in bcc metals below room temperature includes 12 results and, with 4 cases, in which bends on the *D*(*T*) dependences were registered indicating the quantum diffusion process, the results on potassium are among them.

When analyzing the above data, it is established that the main factor that controls the possibility of observation of quantum diffusion of hydrogen in a bcc metal and the relative change of activation energy Δ*Q*/*Q* is its Debye temperature. In earlier studies, such a result was not claimed. It is observed that the bends on the *D*(*T*) dependences occur near the Debye temperatures and can be observed only for metals for which θ_D_ < 350 K. These regularities define requirements to techniques, which can be used to obtain the experimental data on the diffusion coefficients, as well as the list of bcc metals, in which the observation of hydrogen tunneling is possible. All in all, there are 8 metals, and this result is of fundamental importance because tunneling is one of the two existing mechanisms of atomic migration in solids. An additional factor affecting the possibility of observation of quantum diffusion, in our opinion, is the existence in bcc metals of two types of diffusion pathways that differ in equilibrium positions for hydrogen atoms, namely, tetrahedral or octahedral interstices. For pathways involving octahedral interstices, the probability of tunneling is negligible and quantum diffusion cannot be observed. The question on the existence of one or the other pathways in diffusion systems has not been studied so far and the data on tunneling can be used for the determination of a pathway type for protium, deuterium, and tritium in a metal. Moreover, from the regularities stated, it stems that tunneling, that is, the accelerated diffusion in comparison with the classical one, will not be observed at cryogenic temperatures in important engineering metals such as α-Fe, W, Mo, V, and Cr. This result is very important for low-temperature technologies.

## Supporting Information Available

Equipment for assembling and annealing of samples in a vacuum chamber of an accelerator (PDF)
