# Supplementary material for: Debye Temperature and Quantum Diffusion of Hydrogen in Body-Centered Cubic Metals
Source: ACS Omega. 2022 Mar 1;7(10):8385–90. doi: 10.1021/acsomega.1c05902 (PMC8928561; doi:10.1021/acsomega.1c05902)
Supplement: Supplementary file 1 — ao1c05902_si_001.pdf [file ao1c05902_si_001.pdf]

Supporting Information

Debye Temperature and Quantum Diffusion of Hydrogen in Body-Centered Cubic Metals

Vladimir Vykhodets[1*], Olga Nefedova[2], Tatiana Kurennykh[1], Sviatoslav Obukhov[1], Evgenia Vykhodets[3]

[1]Institute of Metal Physics UB RAS
18 S. Kovalevskaya Street, Ekaterinburg, RU 620108
[2]Institute of Engineering Science UB RAS
34 Komsomolskaya Street, Ekaterinburg, RU 620049
[3]Ural Federal University named after the First President of Russia B. N. Yeltsin
19 Mira Street, Ekaterinburg, RU 620002

*Corresponding Author: vykhod@imp.uran.ru

Equipment for assembling and annealing of samples in a vacuum chamber of accelerator

In the as-supplied state, potassium was stored in glass-vacuumed ampules. To break up ampules with potassium, a box with low content of oxygen and water vapor, no more than 0.5 ppm, was engaged (M. Braun Inertgas-Systeme GmbH production). In the box, potassium was transferred into a transport container and placed into a dip of metal plate where flat working surface of the sample was formed; then the box was sealed hermetically with a cap, spring, and vacuum grease (Figure S1(*a*)). Reliable heat and mechanical contact between potassium and metal plate was provided through adhesion. Then, the container was placed into a chamber of accelerating setup, mounted on a holder for isothermal diffusion annealing, and opened under condition of a high vacuum.

Diffusion experiments were conducted in the temperature range from 90 to 260 K. These conditions were provided by means of resistive heater with stabilized power source and massive heat-exchange unit cooled with flowing nitrogen (Figure S1(*b*)), which have a reliable heat contact with the plate and sample. The annealing temperature was changed by varying the heater power. In operation, first, with the switch-off heater, the temperature of the equipment as a whole was set close to that of liquid nitrogen. Then, the heater was switched on and the required temperature was reached. In the course of isothermal annealing, the liquid nitrogen flow and the current of accelerating beam were kept constant within several percent; oscillations of the heat power did not exceed 0.5%. The sample temperature was measured manifold using chromel–alumel thermocouple set near the sample (Figure S1(*b*)), and its deviation from the nominal value did not exceed ± 1 K, which was provided in the main by the constant temperature of liquid nitrogen and large mass of the heat exchange unit. In a special experiment with a reference to which several thermocouples were welded, it was found that the temperature in the irradiated zone of the sample was 3–5 K higher the thermocouple readings. The necessary correction to the temperature of diffusion annealing was taken into account.

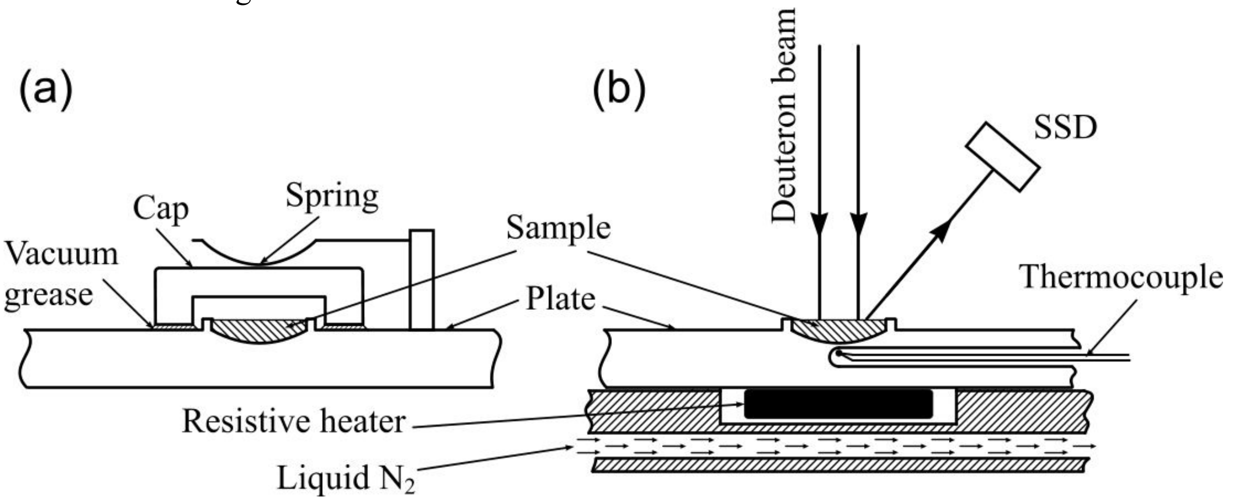

Figure S1. Scheme of sample location in transport container (a) and accelerator chamber (b), SSD stands for silicon surface-barrier detector.
